# Hybrid Ti6Al4V/Silk Fibroin Composite for Load-Bearing Implants: A Hierarchical Multifunctional Cellular Scaffold

**DOI:** 10.3390/ma15176156

**Published:** 2022-09-05

**Authors:** Simone Murchio, Matteo Benedetti, Anastasia Berto, Francesca Agostinacchio, Gianluca Zappini, Devid Maniglio

**Affiliations:** 1 Department of Industrial Engineering–DII, University of Trento, 38123 Trento, Italy; 2 BIOtech Research Center, University of Trento, 38122 Trento, Italy; 3 Lincotek Medical Trento, 38057 Pergine Valsugana, Italy

**Keywords:** lattice structures, silk fibroin, foams, hybrid composites, bone load-bearing scaffolds

## Abstract

Despite the tremendous technological advances that metal additive manufacturing (AM) has made in the last decades, there are still some major concerns guaranteeing its massive industrial application in the biomedical field. Indeed, some main limitations arise in dealing with their biological properties, specifically in terms of osseointegration. Morphological accuracy of sub-unital elements along with the printing resolution are major constraints in the design workspace of a lattice, hindering the possibility of manufacturing structures optimized for proper osteointegration. To overcome these issues, the authors developed a new hybrid multifunctional composite scaffold consisting of an AM Ti6Al4V lattice structure and a silk fibroin/gelatin foam. The composite was realized by combining laser powder bed fusion (L-PBF) of simple cubic lattice structures with foaming techniques. A combined process of foaming and electrodeposition has been also evaluated. The multifunctional scaffolds were characterized to evaluate their pore size, morphology, and distribution as well as their adhesion and behavior at the metal–polymer interface. Pull-out tests in dry and hydrated conditions were employed for the mechanical characterization. Additionally, a cytotoxicity assessment was performed to preliminarily evaluate their potential application in the biomedical field as load-bearing next-generation medical devices.

## 1. Introduction

In recent years, metal additive manufacturing (AM) has made tremendous technological advances, up to the point that functional high-specific metal components can be reliably produced in several industrial fields such as the aerospace and biomedical industries [1,2,3,4]. It is in the latter that metal AM has increasingly grown in interest, to the extent of being considered a potential game-changer for the manufacture of a new generation of prosthetic devices [5,6,7]. In fact, with respect to conventional subtractive or formative manufacturing techniques, the main advantages of metal AM processes (i.e., powder bed fusion (PBF) technologies) are the fast design and manufacture of complex-shaped, customizable (patient-specific) porous structures [8,9]. In this sense, relevant steps can be taken toward a partial or complete mimicking of the morphological structure of bone tissue, and its peculiar hierarchical configuration, using architected cellular materials [10,11,12]. These structures consist of repeating cell units spatially arranged in a distinct pattern. Compared to traditional bulk metal prosthetic devices, they can be set as innovative and versatile platforms to build load-bearing scaffolds that are suitable for bone tissue engineering (TE) applications. The above-mentioned topology-related advantages are also reflected in the mechanical properties of prosthetic devices at a macro-scale level [13,14]. In fact, AM lattice structures exhibit a tunable bone-mimicking mechanical behavior in terms of Young’s modulus and fatigue strength as well as a bone-like mass transport behavior [15,16,17,18]. In particular, the possibility of tailoring its stiffness, reducing the mismatch between the implant and bone tissue, together with the compatibility with new titanium alloys (i.e., β-Ti alloys), guarantee a lower risk of stress shielding, implant loosening, and adjacent bone degeneration [19,20,21,22,23,24,25]. Nonetheless, for a proper prosthetic device design, it is imperative to consider the biological-related features that a bone TE scaffold should have. Among them, osteointegration and bone ingrowth are vital for its functionality and stability, providing a strong mechanical interlocking with the adjacent bone tissue [26]. These issues have been thoroughly studied in the literature and it is well-known that an open interconnected porosity with a specific pore size is a key prerequisite to meet these needs [27,28,29,30]. For instance, Zheng et al. [31] recently reported that strut-based lattice structures (cubic lattices) with an overall interconnected porosity >70% and average pore size of 200 μm exhibited the strongest in vivo osteointegrative capability and implant–bone stability.

However, for such sub-millimetric dimensions, it is necessary to consider the possible thresholds and limitations related to the minimum printable geometrical details as well as to the geometrical accuracy of the printed components [32,33,34]. The morphological precision of the sub-unital elements (i.e., struts and nodes) could be compromised, leading, for example, to an increase in the geometrical defects or severe geometrical imperfections of the struts that negatively affect the fatigue strength and load-bearing capacity of the entire architected cellular structure [16,32,35,36,37].

To overcome these limitations and simultaneously guarantee a proper load-bearing behavior as well as correct bone tissue ingrowth, some hybrid approaches have been proposed in the literature. Among them, coating the metal lattice structure is one of the most explored. For example, Yavari et al. [38] investigated the role of an antibacterial gelatin-based growth factor-loaded coating as a booster for the biological activation of the metal scaffold. Karaji et al. [39] investigated a silk fibroin coating loaded with TCP/vancomycin to prevent implant infections and Li et al. [40] explored it as a potential coating to enhance the osseointegration of titanium lattice structures.

Although a beneficial action of the coating from a biological point of view has been reported in these works, this solution cannot overcome the pore size limitation referred to above of the AM-architected cellular materials.

For this reason, the implementation of an additional structure with tunable porosity and lower pore size range into an AM metal implant might be of further support for bone ingrowth. For instance, the possibility of creating a polymeric-infilled cellular-based lattice device is a potential way to address the open issue above-mentioned. However, to the authors’ knowledge, this architecture has been little explored in the literature [28,41,42,43,44].

In the tissue engineering field, silk fibroin has established itself as a reference material for bone scaffold production due to its assembly ability into different architectures, with tunable morphological and physicochemical properties as well as its noticeable biocompatibility, being a bio-derived natural polymer [45,46]. Silk fibroin scaffolds can be fabricated using different manufacturing techniques such as electrospinning [47], salt-leaching [48], plasma-assisted deposition [49], or foaming processes [50,51,52]. In a previous work by our group, Maniglio et al. [51] described a novel low-pressure method, exploiting nitrous oxide (N_2_O) as a foaming agent, to manufacture silk-fibroin-based scaffolds. The authors demonstrated that due to the high solubility of N_2_O in hydrophilic and hydrophobic substances, the gas can accumulate in different regions of the proteins and that, once pressurized, it allows for the expansion of a silk fibroin solution during its extrusion from a needle. They also revealed that N_2_O does not induce any pH modification of the solution, thus avoiding the gelation of silk fibroin or its early degradation. However, due to the reported poor mechanical properties, foamed scaffolds cannot be considered suitable for bone TE load-bearing applications. Nevertheless, their capacity to fill large cavities, by injecting a stable silk-fibroin bioactive porous filler, allows for an easy combination with the metal AM lattices, for the realization of a hybrid composite system, fulfilling the mechanical and biological requirements that an implant needs. Potential applications of such composite systems can be found in the design and fabrication of a new generation of lumbar spinal fusion cages or for the re-design of titanium endplates for lumbar total disc replacement.

In the present study, we developed a novel hybrid multifunctional composite scaffold consisting of an AM Ti6Al4V cubic lattice structure impregnated with a porous silk fibroin/gelatin foam. The composite material was designed with the aim to add the osteoblast conduction capability to titanium trabecular structures realized by AM that, with regard to fabrication constrains, possessed excessive porosity dimensions for osteoconduction. The research focuses on the optimization of the manufacturing procedures, aiming at optimizing the interaction between the metal structure and the polymeric porous filler made of a silk fibroin/gelatin blend. The polymeric scaffold was obtained by a combination of L-PBF and N_2_O foaming and, for some conditions, an additional electrowetting process. The latter consisted of the simultaneous application of an electric field during the foaming process, inducing a more intimate adhesion with the metal surfaces. Concerning the composite scaffold characterization, an initial rheological assessment of the blended solution was performed, along with the ξ-potential measurements. The characterization of the polymeric foam was conducted by a scanning electron microscopy (SEM) investigation and a statistically based porosity analysis. The evaluation of the polymer–metal adhesive strength was then carried out by performing pull-out tests in the dry and hydrated states. The mechanical properties were then correlated to the failure surfaces and visually inspected by SEM. A final cytotoxicity assessment of the composite was performed to confirm the composite scaffold as a potential candidate for prosthetic applications.

## 2. Materials and Methods

### 2.1. Hybrid Scaffold Preparation

#### 2.1.1. Ti6Al4V Cage Design and Manufacturing

**Figure 1:**
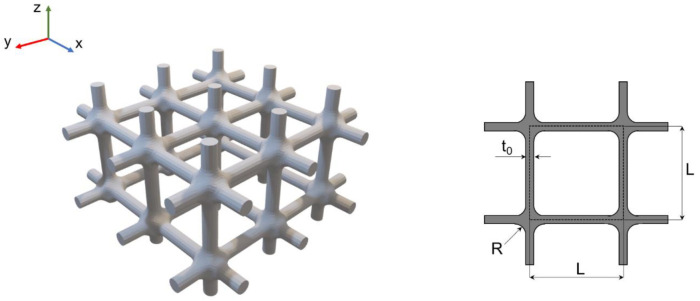
A 3D model of the L-PBF Ti6Al4V simple cubic lattice structure (on the **left**) and the simple cubic lattice unit (on the **right**). T0 is the diameter of the strut (670 µm), R is the radius of the fillet at the junction (600 µm), and L represents the side of the cell unit (4 mm).

Regular cubic unit cell lattice structures were designed as shown in Figure 1, with a nominal strut diameter t_0_ of 670 μm, a strut length L of 4 mm, and a fillet radius R at the junction of 600 μm. The lattice specimens consisted of four unit cells in the XY plane and 1 unit cell along with the sample height (z-axis). This cell architecture was selected due to its simplicity, which facilitates the metrological and biological characterizations undertaken in this work. More complex cell architectures displaying a suitable combination of mechanical properties are left for future work, using the information gained in this work. In particular, it is worth noting that the design space does not have any specific limitations in the maximum size of the scaffold, if not the ones related to the prosthetic device of interest. Limitations in the size might arise if moving toward small lattice unit cells, either in terms of printing quality or correctly infilling the polymeric foam. Indeed, a relatively large cell size was selected to ensure adequate printing accuracy, and therefore, good fatigue performances. More details on the relationship between the cell size, manufacturability, and mechanical properties can be found in [16,25].

The specimens were additively manufactured via laser powder bed fusion (L-PBF) at Lincotek Medical (Trento, Italy), starting from a biomedical grade Ti6Al4V powder, with a particle size in the 15 to 45 μm range. An EOS M290 machine, equipped with a continuous laser with a nominal power of 400 W, was employed. The layer thickness at each manufacturing step was set to 60 μm. Once fabricated, the lattice structures underwent a proprietary heat treatment at a temperature above 800 °C, suitable for the residual stress removal and the transformation of the as-built martensitic α’ microstructure into a more stable α + β lamellar structure. No further surface treatment was performed on the L-PBF specimens, which should therefore be considered in the as-built condition.

The specimens subsequently used for the cytotoxicity assay were cleaned with a proper detergent, rinsed in three consecutive baths filled with demineralized water, dried in an oven at 80 °C, and immediately transferred to a clean room where they were packaged and sealed in Tyvek pouches.

#### 2.1.2. Silk Fibroin and Silk Fibroin–Gelatin Solution Preparation

Silk fibroin (SF) solution was obtained starting from Bombyx mori cocoons (Chul Thai SilkCo., Phetchabun, Thailand). A degumming process was performed to remove the sericin. It consisted of two consecutive treatments in alkaline baths at 98 °C for 1.5 h, respectively, at a concentration of 1.1 g/L and 0.4 g/L Na_2_CO_3_. Once completed, the degummed SF was washed and rinsed multiple times with deionized water (DI) and air-dried overnight. Native SF fibers were then dissolved at a concentration of 20% *w/v* in a 9.3 M aqueous solution of lithium bromide (LiBr) at 65 °C for 4 h. Consequently, to remove LiBr, the solution was dialyzed against DI water for 3 days at room temperature (RT) in a 3.5 kDa dialysis tube (Spectra/por 3 dialysis membrane, Spectrum Laboratories Inc., Piscataway, NJ, USA) with daily water changes, and finally filtered to remove impurities or solid residues in suspension. The silk fibroin solution concentration was measured using a Nanodrop ND-1000 Spectrophotometer (Marshall Scientific, Hampton, NH, USA) and then adjusted to 5% *w*/*v* by dilution with DI water.

Gelatin (G) solution was obtained by dissolving gelatin powder from porcine skin (Type A, gel strength ~300 g Bloom, Sigma-Aldrich, USA) into DI water to reach a final concentration of 20% *w*/*v*. The process was carried out in an oven at a constant temperature of 60 °C, where the solution was kept until use, to avoid the sol–gel transition. The blend solution of gelatin and silk fibroin (G/SF) was obtained by directly mixing the two solutions at a constant temperature of 40 °C under stirring. For the initial rheological evaluation, three different volume ratios were considered, namely G/SF of 1:4, 1:3, and 1:2.

#### 2.1.3. Preparation of the Hybrid Composite Scaffold

Herein, the two manufacturing techniques involved in the production of the metal–polymer composite systems are reported. Namely, the foaming process is reported in Section Gas Foaming, while electrowetting is described in Section Electrowetting. The produced and investigated composite specimens are as follows:

- **SF**: L-PBF titanium cage + 5% silk fibroin foam.
- **SF_EW**: L-PBF titanium cage + 5% silk fibroin electrowet foam.
- **SFG**: L-PBF titanium cage + 5% silk fibroin-gelatin foam.
- **SFG_EW**: L-PBF titanium cage + electrowet foam with 5% silk fibroin–gelatin.

##### Gas Foaming

The foaming procedure reported in the work of Maniglio et al. [51] was adopted to realize the gelatin–silk fibroin foams. Briefly, SF or G/SF solutions were poured into a 0.5 L stainless steel siphon (ICO, Whip it, Vancouver, Canada) equipped with a purging nozzle (inner diameter 10 mm, length 50 mm) and pressurized with N_2_O at a pressure of 10 bar. After vigorous shaking and a subsequent resting stage of at least 10 min, to guarantee the proper gas dissolution, the solution was extruded into the metal cage. Due to the gas expansion, the SF or G/SF solutions could be directly extruded in a foamed state throughout the purging nozzle. The foamed metal cage was immediately soaked and frozen in liquid nitrogen to preserve the foam morphology and avoid its collapse. Subsequently, the hybrid scaffold underwent a lyophilization process at −50 °C for 72 h, guaranteeing a stable drying process and complete N_2_O removal.

Due to their instability in the water solution, the samples underwent a post-processing stabilization stage in a pure methanol solution for 10 min and were then rinsed three times, for 10 min, in a pure bi-distilled water bath. As stated by Maniglio et al. [51], methanol exposure leads to the formation of a more crystalline structure due to an increase in the β-sheet network (up to 30%), which improved the stability of the foam in water solution.

##### Electrowetting

The electrowetting procedure consisted of a combination of the foaming process above-mentioned with a simultaneous application of a low voltage to the metal cage. 

**Figure 2:**
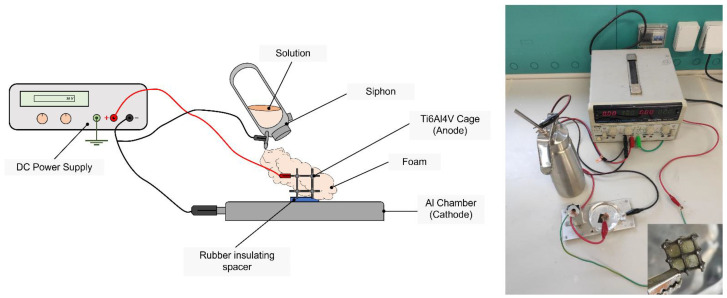
An illustration and image of the electrowetting setup, composed of a DC power supply, a siphon, a counter-electrode chamber in aluminum, and the Ti6Al4V cage, acting as the anode of the electrophoretic chamber. A close-up image of an electrowetted composite system is reported in the lower-right corner of the figure.

The apparatus, depicted in Figure 2, was composed of a direct current (DC) power supply connected to the siphon nozzle and an aluminum deposition chamber, acting as counter-electrodes, and to the L-PBF titanium cage, acting instead as the positive electrode. The applied voltage was set at 30 V. The DC power supply was turned on just before the foam extrusion throughout the nozzle and kept for a total time of 1 min. The voltage–time application was set to guarantee a sufficient interaction between the foam solution and the metal cage under the electric field but still avoid foam collapse. Once the electrowetting was completed, the metal–polymer system underwent liquid nitrogen freezing, subsequent lyophilization, and methanol-based stabilization, as described in the previous section.

### 2.2. Hybrid Composite Scaffold Characterization

#### 2.2.1. Rheological Tests

Rheological characterization of the G/SF blend solution was carried out to investigate the sol-gel transition temperature of blend solutions with different G:SF ratios. Rheological tests were performed using an HR-2 Discovery hybrid rheometer (TA Instruments, DE, New Castle, USA) equipped with a cone plate geometry (diameter 50 mm, 2° cone plate angle, and truncation gap of 100 μm) and an aluminum Peltier plate. The analyses were performed in temperature sweep mode at a constant angular frequency of 1 Hz and 1.0% strain. The temperature range of interest was between 50 °C and 15 °C, with a cooling ramp rate of 1.0 °C/min. A soak time of 60 s at 50 °C was imposed before the beginning of each analysis. Three replicates for each inspected solution were considered to have sufficient statistical reliability. The sol-gel transition temperature was measured at the intersection of the modulus curve, namely the storage (G′) and loss (G″) modulus. The average values over the three replicates were then considered.

The rheological-inspected solutions are herein reported. Blended solutions were obtained, keeping the G/SF system at a constant solution concentration.

- **G:** pure aqueous gelatin solution at 20% *w/v* concentration.
- **G/SF 1:2:** blend solution of gelatin and silk fibroin in a ratio G:SF of 1:2.
- **G/SF 1:3:** blend solution of gelatin and silk fibroin in a ratio G:SF of 1:3.
- **G/SF 1:4:** blend solution of gelatin and silk fibroin in a ratio G:SF of 1:4.

#### 2.2.2. ξ-Potential

The electrophoretic motion of the SF and G proteins and their blend were evaluated by a ξ-potential measurement. A Malvern Zetasizer Nano-ZS particle analyzer was used to carry out the measurements. A total of 48 ξ-potential measures, divided into four tests per solution, were performed to guarantee enough statistical reliability. Each solution was prepared at the previously described concentrations and afterward diluted in DI water by a factor of 100 times. All tests were performed at 40 °C to avoid undesired gelation, with an initial equilibration time of 60 s and a subsequent 15 s equilibration time between each measure.

#### 2.2.3. Scanning Electron Microscopy (SEM)

The morphology of the composite metal–polymer system and the foam adhesion to the metal cage were inspected using a Supra 40 Field-Emission Scanning Electron Microscope (Zeiss, Germany). Images were acquired in secondary electron mode at 5.00 kV for macroscopical evaluation of the different composite manufacturing techniques, pure foaming, and electrowetting, while for the analysis of the foamed single strut and its adhesion, a 2.00 kV was imposed. Before the SEM inspection, specimens underwent the sputtering of a platinum-palladium (Pt/Pd, 80:20) conductive thin coating (Q150T ES, Quorum Technologies, Lewes, UK).

#### 2.2.4. Porosity Analysis

**Figure 3:**
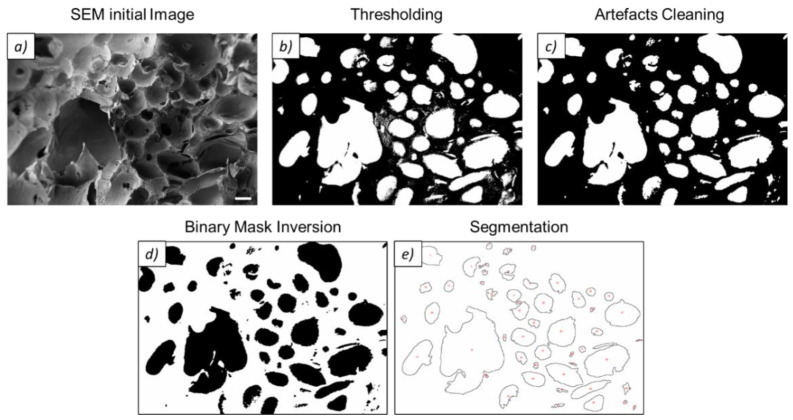
The workflow of the pore analysis in Fiji. Starting from the SEM initial image (**a**), thresholding was applied with the percentile method (**b**). Subsequent artifact cleaning (**c**) and binary mask inversion (**d**) were applied before the segmentation process (**e**). Detected porosity along the frame edges was excluded.

The SEM images were also used to investigate the pore dimension of the four different foam categories and to verify the impact of the production process. Five micrographs for each of the four conditions were analyzed with Fiji open-source software. The image analysis was carried out following the steps reported in Figure 3. Briefly, FE-SEM images underwent a thresholding process with the percentile method and a subsequent segmentation after an artifact cleaning, aimed to remove unwanted porosity in the lower region of the distribution. Additionally, pores with an open perimeter at the edge of the image were not considered for the evaluation of pores. As a result of the highly irregular morphology of most pores, the pore area (*A*) was first calculated. The equivalent pore diameter *D_eq_* was then derived from the pore area by the assumption of perfect circularity (*C*). The latter is a dimensionless parameter ranging from 0 to 1, where 0 refers to highly non-circular shapes, while 1 indicates a perfect circle. Circularity can be calculated from the following equation:

(1) $$C=\frac{4\pi *A}{P^{2}}$$

where *A* is the pore area and *P* is the pore perimeter.

For the porosity assessment of the foams, an additional shape factor, the aspect ratio (*AR*), was inspected. This is an index of how elongated the pore is and it can be defined as the ratio between the maximum and the minimum distance of the pore, as reported in Equation (2):

(2) $$AR=\frac{l_{MAX}}{l_{MIN}}$$

where *l_MAX_* and *l_MIN_* are, respectively, the maximum and minimum Feret pore distances.

The main descriptive statistic parameters were then evaluated such as the mean value, the median, the standard deviation, quartiles (Q1 and Q3) as well as the interquartile range (IQR). Additionally, to perform a comparative study among the four conditions of interest, a one-way ANOVA test followed by a multi-comparison Tukey’s test was performed. The confidence levels were assigned as follows: *p* ≤ 0.1 (.), *p* ≤ 0.05 (*), *p* ≤ 0.01 (**), *p* ≤ 0.001 (***).

#### 2.2.5. Pull-Out Tests

Pull-out tests were performed to evaluate the metal-polymer adhesion, considering a different specimen design just made of a foamed metal strut. The metal strut acts as the fiber, whereas the SF or G/SF foam acts as the matrix. The rationale behind the specimen design is to guarantee a free end of the metal strut that can be constrained and gripped to a tensile machine. For this reason, to guarantee proper placement on the testing machine, the sample was fixed at the bottom of an aluminum chamber with tape to avoid any detachment during handling. The aluminum foil also acts as a counter electrode for the electrowet specimens (SF_EW and SFG_EW). Pull-out tests were carried out using a Bose Electroforce 3200 machine (TA Instruments, New Castle, DE, USA) equipped with a 200 N load cell (sensitivity of 0.05 N) and stainless-steel grips. All tests were performed at a constant loading rate of 0.02 mm/s. Five replicates for each of the four conditions were tested either in their dry state or in wet conditions. Wetted samples were soaked in DI water before the test and kept hydrated during the pull-out test by a dropwise addition of DI water.

To measure the degree of adhesion of the foam to the metal cage, the apparent shear strength was derived from the force-displacement curve, according to the Kelly-Tyson equation, as described in the work of Greszczuk et al. [53] and Viel et al. [54] and reported herein.

(3) $$\tau_{app}=\frac{F_{deb}}{\pi D_{f}L_{embf}}$$

where $\tau_{app}$ is the apparent interfacial shear strength, and $F_{deb}$ the debonding force (considered as the maximum force value of the force-displacement curve). $L_{embf}$, instead, represents the length of the fiber, which is embedded into the matrix foam, and it is equal to 2.5 mm. $D_{f}$ is the fiber diameter, which in this context is equal to the strut diameter. It is worth noting that an average diameter of 700 μm was considered, rather than the nominal one of 670 μm. The average value was taken from the work of Dallago et al. [16], where the authors metrologically investigated cubic lattice structures with the same cell units considered herein. The decision to choose the average diameter length was related to the relevant discrepancies in dimensions among the printed struts and their discrepancies with the input CAD model, typical of AM products. Additionally, an average strut thickness can also partially take into account the profile texture of the as-built surfaces. It is, however, important to point out that the effect of interfacial friction and stable fiber debonding could not be fully considered with the above-mentioned model, as also described in the work of Sørensen et al. [55]. Being aware of these possible limitations, the authors focused on a comparative study among the four sample categories, particularly on the effect of the processing technique, leaving for future works and developments a deeper quantitative analysis of the adhesive behavior of the foam to the L-PBF strut.

Before the statistical data analysis and the curve averaging, a smoothing procedure was applied to each load-displacement curve, to reduce the data noise. Specifically, a nonparametric local smoothing known as LOESS (Local estimated scatterplot smoothing) method was applied in OriginPro 2018 software (OriginLab Corporation, Northampton, MA, USA). The statistical comparison was performed by adopting the typical descriptive parameters previously defined for the porosity assessment, in Section 2.2.4. Additionally, averaged load-displacement curves with scatter bands were calculated over the five tested specimens, either for the dry or wet conditions.

#### 2.2.6. Cytotoxicity Assay

The cytotoxicity of AM metal cage foam systems was evaluated using human lung fibroblast cells (MRC5). Cells were expanded and cultured in a medium composed of minimal essential medium (MEM, Gibco, Thermo Fisher Scientific, Waltham, MA, USA), 10% inactivated fetal bovine serum (Euroclone, Pero, Italy) with a supplement of 1% L-glutamine (Euroclone, Pero, Italy), sodium pyruvate (Gibco, Thermo Fisher Scientific, Waltham, MA, USA), antibiotic-antimycotic (Euroclone, Pero, Italy), and nonessential amino acids (Sigma Aldrich, St. Louis, MO, USA). Standard conditions (37 °C and 5% CO_2_) were used for the cell culture. The lactate dehydrogenase (LDH) cytotoxicity assay kit (Thermo Fisher Scientific, Waltham, MA, USA) was used to measure the amount of LDH released by cells during their death. The test was carried out according to the European Standard EN ISO-10993-12:2004 and 10993-5:2009, and all tested specimens underwent a sterilization process before testing. It involved a bath in an ethanol solution (70% *v*/*v*), followed by two consecutive washes in sterile DI water. The experiment was performed on bare L-PBF Ti6Al4V cages, the four metal–polymer composite structures (see Section 2.1.3) and on the SF and SFG foams as comparative control specimens.

Briefly, all samples were incubated for 72 h without cells in a medium in the absence of phenol red and with heat-inactivated serum (conditioned medium). After the incubation, the conditioned medium was deposited onto MRC5 cells at 70% of confluence. Cells were previously seeded in a 96-well plate and incubated for 48 h. The cell density seeded was equal to 5000 cells/well. Positive (CTRL+) and negative (CTRL−) controls were characterized by fully lysate cells and cells cultured in standard medium. 

After their incubation, all samples were prepared according to the manufacturer’s instructions. The amount of LDH released in the medium was then measured through a Tecan Infinite 200 microplate reader (Tecan Group, Männedorf, Switzerland). The background wavelength was recorded at 680 nm, whereas the absorbance was detected at 490 nm. Average values, along with their standard error, were evaluated on five replicates for each condition, according to the statistical theory of error propagation.

## 3. Results

The current section first reports the results of a preliminary rheological characterization of the SF-G blended solution at different G:SF volume ratios, with the aim of investigating the most suitable solution for the foaming and electrowetting processes. The electrophoretic behavior of the proteins in an aqueous solution was deduced on the base of the ξ-potential measurements. Second, a morphological investigation of the foams in the four different conditions (described in Section 2.1.3) was then carried out by FE-SEM, with a particular focus on the porosity analysis. The polymer–metal adhesion was then inspected by employing a pull-out test and a further visual inspection of the foamed single struts. Finally, the cytotoxicity behavior of the metal–polymer system is reported and discussed.

### 3.1. Preliminary Solution Characterization

#### 3.1.1. Rheological Tests

**Figure 4:**
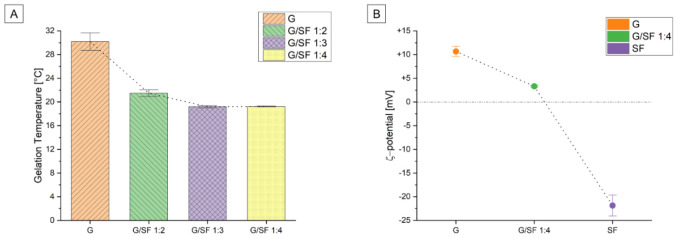
(**A**) The bar plots of the mean gelation temperatures with respect to the solution composition are reported along with the error bars. The dotted line indicates the trend connection line of the temperature mean values. The investigated solutions were pure gelatin (G) and the mixture of gelatin/silk fibroin in different volume ratios, respectively, of 1:2, 1:3, and 1:4. (**B**) The ξ-potential interval plot showing the mean value and the SD error bar of pure gelatin (G), G/SF 1:4, and pure silk fibroin (SF) is reported with the mean trend line.

The rheological investigation revealed that increasing the amount of aqueous SF solution in the blend led to a decrease in the sol-gel temperature. This was particularly evident moving from a pure G solution, which exhibited a gelation temperature of about 30 °C to the G/SF 1:2 solution. The T_sol-gel_ indeed decreased by about 9 °C from the gelation temperature of the pure solution, as reported in Table 1. A further decrease in the G content in the blended solution still induced a temperature drop, even if less evident with respect to the previous condition, until a possible stationary level of T_sol-gel_ equal to 19 °C for the blended solutions G/SF 1:3 and G/SF 1:4, as visually represented in Figure 4A.

As reported by Gil et al. [56], the introduction of SF into the pure G solution leads to a reduction in the available triple-helical crosslinking G sites, facilitating network dissolution at lower temperatures.

A lower sol-gel transition temperature is beneficial for the foaming process to avoid any unwanted transition to gel before the foam extrusion through the nozzle. This is fundamental to properly wet the metal-cage surface and correctly infill the cavities of the cubic lattice structure and, therefore, for the correct manufacturing of the composite system. For these reasons, the authors ended up choosing the G/SF 1:4 solution as the preferred blend composition, with the intent of hindering the physical gelation of the extruded foam close to room temperature. However, it should be noted that the mixed solution was precautionarily stored at 40 °C before the foaming process, which guaranteed the liquid state of the solution and a safety time before the solution cooling close to the gelation temperature.

#### 3.1.2. ξ-Potential

The electrowetting process used to fabricate the SF_EW and SFG_EW consists of a combined technique of foaming and electrophoretic deposition (EPD). Therefore, it is fundamental to investigate the electrophoretic motion of silk fibroin and gelatin in an aqueous medium under the application of an electric field. The motion of charged particles and their deposition onto a titanium surface is indeed at the base of the electrowetting process. For this reason, the ξ-potential of the G, SF, and G/SF blend solutions was calculated. The measured data are summarized in Table 1 and visually schematized in Figure 4B. The pure gelatin solution was positively charged with a ξ-potential of about +11 mV in an aqueous solution. Silk fibroin was instead negatively charged, reporting a ξ-potential of about −22 mV still in an aqueous solution. When gelatin was mixed with the silk fibroin solution in a volume ratio of 1:4, the ξ-potential decreased to +3 mV compared to the gelatin (+11 mV). It is therefore possible to state that the negative charges of silk fibroin are balancing the positively charged gelatin chains, forming a polyelectrolyte complex through electrostatic interactions.

### 3.2. Polymer–Metal Interface Evaluation

**Figure 5:**
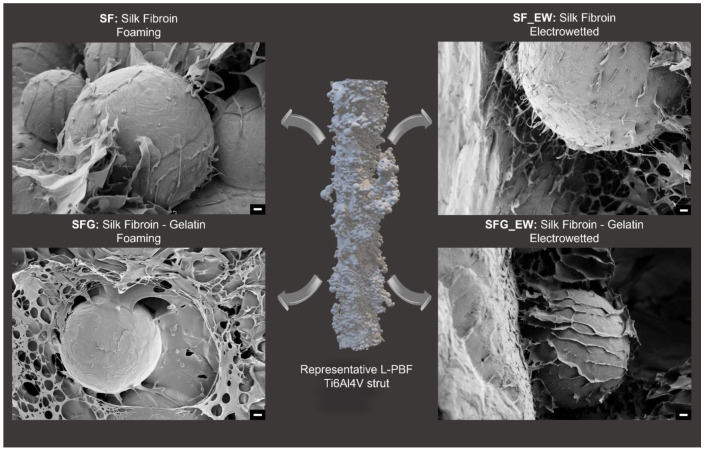
A representative close-up of the FE-SEM images of the four composite specimens at the metal-polymer interface were attached with a representative 3D reconstruction of a single strut of the LPBF Ti6Al4V lattice structure. The four FE-SEM images represent the silk fibroin foamed (SF) composite specimens (top-left), the silk fibroin electrowet (SF_EW) composite specimens (top-right), the silk fibroin/gelatin foamed (SFG) composite specimens (bottom-left), and the silk fibroin/gelatin electrowet (SFG_EW) composite specimens (bottom-right). Scale bars were set to 2 µm for all four images.

An initial evaluation of the interaction between the polymeric foam and the L-PBF Ti6Al4V struts was performed using the scanning microscopy technique. Particularly, the polymeric structures around unmelted titanium particles were investigated by collecting FE-SEM close-up images. In Figure 5, exemplary images of the polymer/metal interface are reported, along with a representative image of a strut of the lattice. Pure SF foams reported partial coverage of the strut particles, alternating thin lamellar structures, wrapping the outermost metal surface, to agglomerates of the plate-like SF leaves. When compared to the SFG foams, the role of gelatin in the blend solution was clear. The addition of G led to a reduction in the lamellar-like structure typical of SF and the formation of a more pronounced porous structure. This result agrees with what was reported by Lu et al. [57], where SF/G scaffolds were investigated. The authors stated that an increasing content of G in an aqueous SF solution guarantees higher stability of the structures against water solubility, thus allowing for the formation of pores after the lyophilization process. Additionally, as visible from the SFG image, the binding effect of G was evident: a more intimate connection with Ti6Al4V particles was indeed noticeable with thicker and larger portions of the titanium particles covered by the polymeric foam.

**Figure 6:**
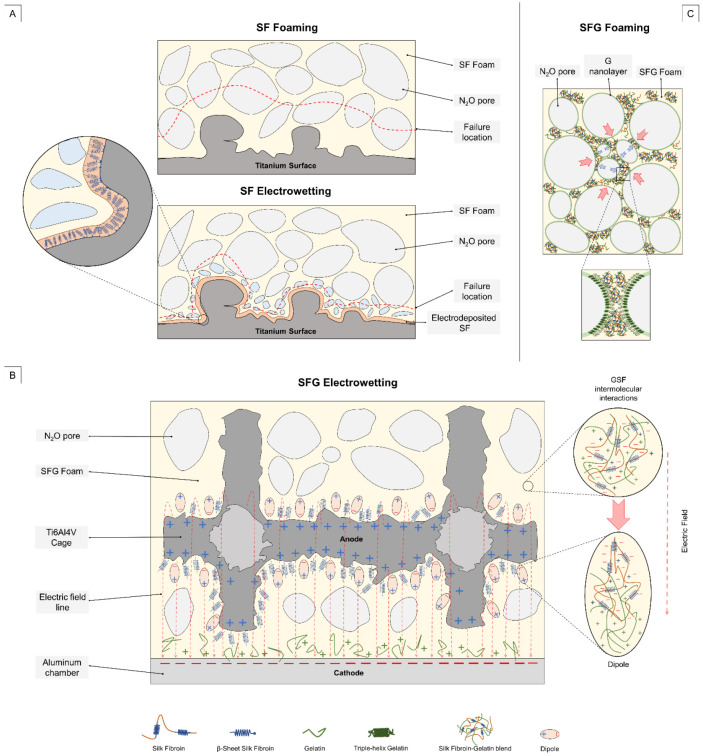
Sketches of the proposed mechanisms related to the foaming and the electrowetting processes. (**A**) The drawing depicts a comparison between the foaming and electrowetting of a pure silk fibroin solution, highlighting the electrodeposition of SF and its reorganization into a more ordered structure and β-sheets enriched the coating of the outermost titanium surface. Possible different fracture location lines, ascribable to the role of hydrolysis-induced porosity, are reported. (**B**) The sketch reports the main possible driving mechanisms of the SFG electrowetting. The intermolecular interactions of SF and G in the blend solution generated polyelectrolyte complexes, which were reordered along the electric field lines and reorganized in dipole configurations. The latter exposed the negative chains toward the titanium surface, while the positive side faced the counter-electrode. (**C**) The SFG foaming sketch highlighting the role of gelatin in possibly creating a gel nanolayer at the liquid–gas interface, wrapping the N20 particles. Combined with the G/SF complexes, this can help hinder Ostwald ripening or coalescence phenomena.

Moving to the electrowet specimens, SF_EW foams tend to form a gel-like coating on the outermost titanium surface. This adhering coating is generated due to the polyampholyte behavior of silk fibroin, which alternates anionic and cationic side chains. Because of the prevalent negative charge that SF assumes in an aqueous solution, once the protein is subjected to an electric field, it shifts toward the anode of the electrowetting system. Therefore, protein accumulation on the titanium surface is possible by exploiting the same principle of the anionic electrophoretic deposition (EPD) of SF, as explained in [58,59]. In contact with the metal surface, a rearrangement of the SF structure occurs, with a β-sheets reorganization, causing the formation of anchoring fibrils and beads (see Figure 6A). As visible in the FE-SEM image, porosity is also detectable and must be imputed to the combined action of the porogenic gas, the hydrolysis induced by the electric field, and the freeze-drying process. Some larger plates between fibrils and beads have also been noticed, in agreement with the work of Maniglio et al. [58].

Electrowet SFG specimens exhibited a more uniform and intimate coating when compared to the SF_EW and the SFG foams. With respect to SF_EW, even though still noticeable, a lower number of nanofibrils acting as anchoring points were visible on the Ti6Al4V particles. In contrast, dense leaf-shaped structures fully wrapped the metal outermost surface. This behavior might be related to the reorganization of the two polymers in an aqueous solution under the electric field due to the intramolecular interactions between the polymer chains such as the electrostatic bonding of ions and dipoles and dipole van der Waal forces [60]. Particularly, despite the neat charge of the G-SF aqueous solution being positive, not only G-SF complexes are formed in the blend solution, but it is also reasonable to consider the presence of protein chains of G and SF that are not intermolecularly linked. SF chains still move toward the anode such as in the SF_EW specimens, while G chains are more prone to deposit at the cathode due to their predominant positive charges. Instead, G-SF complexes might be spatially reorganized in a dipole configuration along the electric field lines, with G chains facing the aluminum plate and SF chains closer to the titanium surfaces of the cage (see Figure 6B). This electrically driven spatial reorganization might explain the lower presence of nanofibrils onto the outermost titanium particles than the SF_EW specimens. Additionally, this reorganization of G–SF complexes, self-assembling into leaf-like structures alternated to microfibrils, drastically changes the morphology when compared to the more circular porous net structures of the SFG foams.

### 3.3. Porosity Assessment

**Figure 7:**
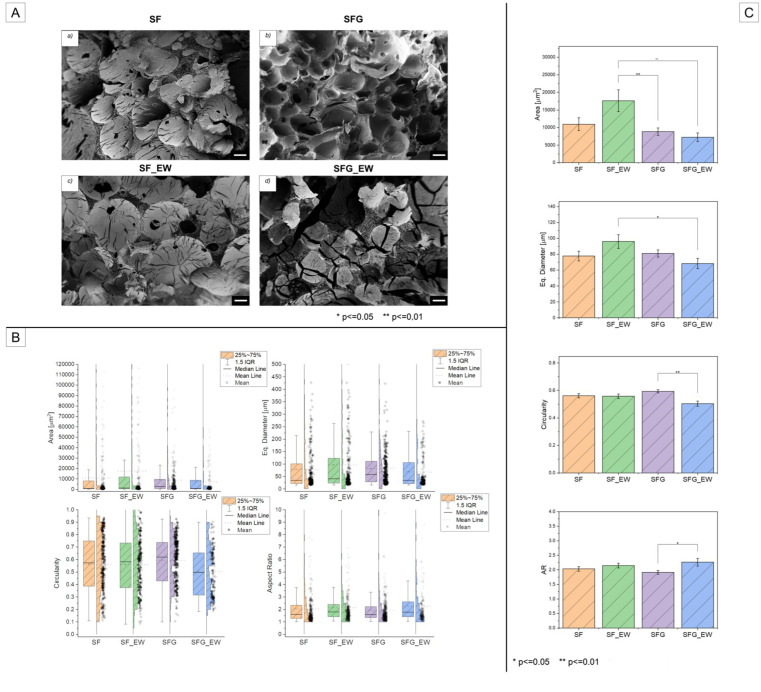
(**A**) Example of FE-SEM images of the four different foams involved in the porosity analysis. The scale bar was set to 100 µm. (**B**) Box plots with the distribution histograms for the four porosity parameters investigated, namely, the area (μm^2^), the equivalent diameter (μm), the circularity C, and the aspect ratio AR. The box charts report the IQR, mean, and median values. The upper and lower bounds were set at 1.5 IQR. (**C**) Multi-comparison bar plots with the *p*-value significance were reported for the area, equivalent diameter, circularity, and aspect ratio (AR).

Figure 7A reports a set of FE-SEM images for each of the four tested conditions. These images depict a central area of each foam located in the cage porous region. For all the conditions, some cracks were clearly visible in the inner part of the pores and in some cases, connecting multiple pores. This can be ascribed to the shrinking that occurs during the freeze-drying stage, which to some extent modifies the porous structure either at a macro scale (cracks) or at a microscale level, introducing a secondary smaller porosity. FE-SEM images were exploited to carry out the pore analysis investigation, as reported in Section 2.2.4. The main results of this analysis were then summarized in Table 2, where the mean, standard deviation, median, and IQR values are reported for the four inspected conditions. It is, however, worth noting that we limited the porosity analysis to only the FE-SEM investigation. We are aware that this might be a limitation, particularly in controlling the degree of interconnectivity of the foam porosity, nonetheless, this decision can be mainly ascribable to two reasons. The first is related to the nature of the composite material itself, which does not allow or makes it extremely challenging to carry out techniques such as computed tomography (CT) scans or gas pycnometry. The second is, instead, related to the fact that Maniglio et al. [51] had already deeply investigated the morphological features including the porosity assessment of the stand-alone polymeric foams by CT scans and the n-hexane porosity assessment. As visible from the boxplots in Figure 7B, the distribution of D_eq_ was not symmetrical and was highly skewed for all four conditions. As reported in Table S1*,* the SF metal foam samples exhibited a skewness value of 2.12, SF_EW of 2.05, while there was an SFG and SFG_EW of 1.80 and 1.41, respectively. Additionally, the excess kurtosis of the four distributions was calculated, revealing a leptokurtic behavior of the distributions (see Table S1). The right-skewed distribution, together with a leptokurtic behavior (kurtosis higher than 3, see Table S1), suggests the presence of a heavy tail and a higher number of outliers toward larger pore diameters. The major number of pores (50% of the dataset) instead resided in the IQR range, which shifted more toward smaller diameters. Indeed, for all of the above-mentioned conditions, most of the pores showed an equivalent diameter D_eq_ lying between 80 and 100 μm, with a mean value of 77.7 μm, 96.1 μm, 81.1 μm, and 68.4 μm, namely for the SF, SF_EW, SFG, and SFG_EW metal-foam specimens, respectively.

As previously discussed, it is also worth focusing on the range between Q3 and the maximum value, where 25% of the data resided. This range showed pore diameters that were considerably larger than 100 μm. Indeed, the maximum values of 543 μm, 620 μm, 422 μm, and 271 μm were reported for the SF, SF_EW, SFG and SFG_EW conditions, respectively (see Table S1). It is possible to state that for all the tested conditions, a broad range of pores can be generated, suggesting the stochasticity of the foaming process. Nonetheless, it is worth noting that this wide porosity range is extremely beneficial from a biological point of view, specifically in terms of osseointegration. As reported in [61,62], either the micro-porosity (lower bound of the distributions) or large pores (tail of the skewed distributions) played a crucial role in bone ingrowth. Large pores (i.e., a pore diameter of about 200–300 μm) are fundamental for the initial cell adhesion and proliferation of osteoblasts on the scaffold surface, while microporosity is also necessary for the nutrients to flow, for scaffold vascularization, and its permeability.

With respect to porosity, their morphology also needs to be considered and evaluated. For this reason, two shape factors, circularity (C) and aspect ratio (AR), were considered. Additionally, in this case, the skewness and kurtosis of the distributions were calculated, as reported in Table S1. The circularity reported a wide symmetrical distribution, with skewness values close to 0 (−0.17 for SF, −0.01 for SF_EW, 0.18 for SFG, and 0.18 for SFG_EW) and a platykurtic behavior, with the excess of kurtosis always negative. The mean values of the four conditions always lay in the 0.5–0.6 range, while the IQR was between 0.3 and 0.4. The AR dataset, calculated for the four different classes of samples, reported a highly positively skewed distribution with leptokurtic behavior. The mean AR values were 2.03 for SF, 2.14 for SF_EW, 1.91 for SFG, and 2.26 for SFG_EW. These values, together with the IQR (see Table 2), suggest that most of the pores exhibited a slightly elliptical shape with an AR higher than 1. Despite the shape-factor distributions, again providing an indication of the randomicity of the process, it is possible to state that porosity is reasonably regular and uniform in shape. This is another key factor in properly promoting osteointegration, indeed, as reported in the literature [63], a regular pore is a preferential site for cell bridging during the proliferation stage.

In Figure 7C, the box charts of the previously discussed pore parameters, together with the *p*-value statistical significance (asterisks), are reported. This allows the material to be controlled (in terms of gelatin addition) and the effect of the electrowetting (in terms of applied voltage) onto the pore dimensions and morphology. Concerning the pore dimensions, and particularly D_eq_, there was no evident significant effect of the gelatin addition during the foaming process, while comparing SF_EW and SFG_EW, a very significant difference (*p*-value ≤ 0.01) was seen. It is worth noting that a statistically relevant difference was also reported when comparing the area mean values of the SF_EW and SFG specimens.

It is therefore reasonable to state that the gelatin addition partially impacts the foam porosity, but this effect is even more evident in the presence of an electric field. The combined effect of voltage application and gelatin addition seems to induce a more pronounced decrease in the pore dimensions with respect to the SF_EW composite specimens.

Gelatin is a well-known binding and emulsifying agent [64], which guarantees high stability of the foams. Due to its slightly relevant surface activity, typical of water-soluble proteins, it can form a nanometric adsorption layer at the liquid-gas interface, where the protein tends to concentrate, promoting its gelation [65,66]. The addition of gelatin to the SF solution might reduce the Ostwald ripening and coarsening phenomena, which leads to the formation of pores of larger areas, as measured for the SF_EW composite specimens. Gelatin might indeed assemble to form an adsorption layer around the gas particles and (or) simultaneously electro-sterically hinder the collapse of adjacent pores, as schematized in Figure 6C. Interestingly, comparing the maximum values of areas (or D_eq_ as previously reported), a decreasing trend moving from SF_EW to SFG_EW is noticeable. Additionally, the skewness of the distributions as well as the excess of kurtosis tended to decrease, with an evident drop for the SFG_EW specimens. These trends indicate that a lower number of outliers were detected for the SFG foams, with either or without electrowetting. The additional and more evident drop, related to SFG_EW specimens, can be addressed by the voltage application. A possible hypothesis can be related to an additional spatial constraint given by the electric field, which limits or slows down the foam expansion. This might be ascribed to two different phenomena, which might simultaneously occur during the foam extrusion. First, the SF and G polymer chains tend to alter their initial configuration, reorganizing into more ordered configurations [56,58,59,67]. Second, the electric field guarantees a better and more intimate adhesion of the SF-G blend solution to the Ti6Al4V struts, as discussed in more detail in the next section. 

Moving to the shape factor analysis, for C or AR, a statistically significant difference was evaluated among the SFG and the SFG_EW specimens. Pore circularity decreased moving from the SFG to the SFG_EW conditions, thus leading to more elliptical pores and consequently inducing an AR increase. The morphological change in the pores can be ascribed to the above-mentioned effects attributed to the electric field. The electrically induced spatial constraint of the foam, particularly close to the strut surfaces, might lead to a pore stretching due to a local increase in the stiffness or gelation of the solution at the liquid-gas interface. It should be noted that this decreasing trend of C was not evident compared to the SF and SF-EW compounds, suggesting that G could undergo a more pronounced spatial reorganization of random coils into triple-helix configurations, rather than the SF β-sheet reorganization.

### 3.4. Pull-Out Test

In this section, the adhesion behavior of the four different foams onto the titanium struts is investigated. For the sake of clarity, in the following discussion, the authors decided to first focus on the pure silk fibroin composite scaffolds manufactured by pure foaming or electrowetting and tested in a dry state. Subsequently, the comparison with the SFG and SFG_EW specimens, still in a dry state, was carried out, particularly by analyzing the role of G addition as well as the effect of an electric field on the foam-metal adhesion. The pull-out results in hydrated conditions were then evaluated and a comparison between dry and wet adhesion is discussed.

**Figure 8:**
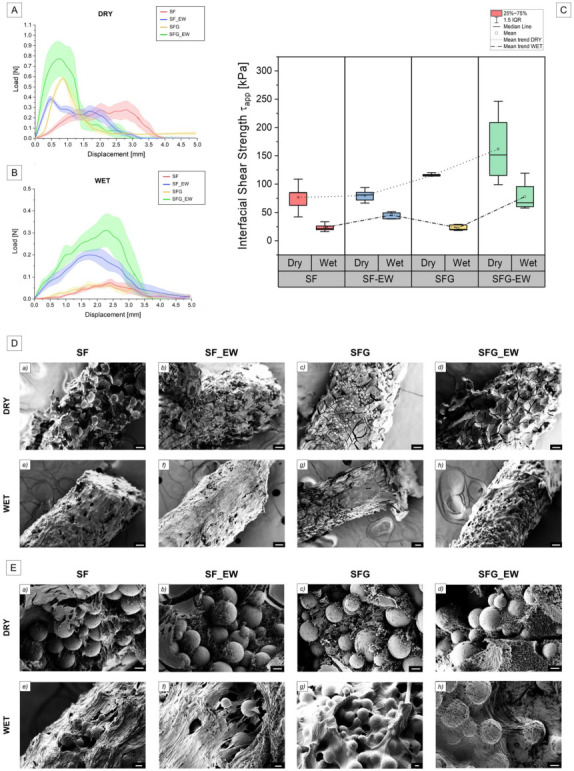
The averaged force–displacement curves pull-out curves for dry (**A**) and wet (**B**) conditions for the four investigated classes of specimens. Scatter bands are also represented. (**C**) Comparative boxplots, depicting the interfacial shear strength values against the four different classes of specimens for the two different testing conditions (dry and wet). Boxplots show the IQR, mean, and median values. The upper and lower bounds were set at 1.5 IQR. Dotted and dashed lines represent the mean trend line for dry specimens and the mean trend line for wet specimens, respectively. (**D**) The FE-SEM images of the pull-out failure surfaces for the dry (**a**–**d**) and wet (**e**–**h**) specimens. The scale bars were set to 100 μm. (**E**) Close-up FE-SEM images of the pull-out failure surfaces for the dry (**a**–**d**) and wet (**e**–**h**) specimens. Scale bars were set to 10 μm.

Figure 8A,B report the averaged load–displacement curves of the four tested configurations under dry and wet conditions. The debonding forces, *F_deb_*, and the apparent interfacial shear strengths, *τ_app_*, were calculated from the pull-out tests, as reported in Section 2.2.5. The average values, along with their standard deviations, are summarized in Table 3. For the dry pull-out tests, the mean *τ_app_* values were 76.7 kPa, 79.8 kPa, 115.1 kPa, and 162.1 kPa, for the SF, SF_EW, SFG, and SFG_EW foams, respectively. A clear increasing trend in the dry interfacial shear strength was noticeable in Figure 8C, moving from the pure SF to the SFG_EW foam. 

Considering the pure SF solution, only a small and no statistically significant difference was evident between the mean *τ_app_* values of the conditions SF (76.7 kPa) and SF_EW (79.8 kPa) conditions. Although this might suggest that the electrowetting of silk fibroin does not effectively improve shear adhesion, it is also important to evaluate their pull-out failure surfaces. Two potentially different failure mechanisms of the foam (composite matrix of the system) seem true. SF foams appear to report a cohesive failure, meaning that the failure occurs in the matrix rather than at the metal–polymer interface (Figure 8D(a)). The SF_EW specimens, instead, exhibited an interfacial failure, locally alternating adhesive and cohesive debonding regions (Figure 8D(b)). As reported in [68], cohesive failure at the polymer–metal interfaces should be preferable to obtain better coating adhesion. However, when focusing on close-up FE-SEM images (Figure 8E(a,b)), the residual SF_EW foam appeared in a greater amount and was more adhered to unmelted Ti6Al4V particles compared to the pure foam samples of SF. The electric field not only induces the formation of SF nanofibrils (see Figure 5), and thus a more intimate adhesion, but it also guarantees a higher protein mass transport at the metal interface. Therefore, SF proteins can be more densely packed along with the strut texture profile, properly filling the asperities and cavities typical of L-PBF as-built surfaces. To draw a hypothesis on the different failure behavior, the authors believe that it is also necessary to consider the hydrolysis-induced porosity generated by the electric field (see Figure 6A for hypothetical failure mechanism sketch). This porosity should be generally smaller in size with respect to the one induced by the N_2_O foaming gas. Capillary forces might drive small gaseous pores toward the metal surface in the proximity of titanium surface particles. Concerning SF foams, pores were generated just by the porogenic foaming gas, reporting larger dimensions compared to the L-PBF titanium particle sizes (see Table 2). N_2_O pores might therefore preferentially settle onto the outermost surfaces of protrusion, being incapable of deeper penetration into the Ti6Al4V particle cavities and in closer contact to the strut surface. Consequently, under tension, the critical failure location of the SF specimens shifted further away from the actual titanium surface, leading to a failure such as the FE_SEM image in Figure 8D(a). 

The addition of gelatin (SFG specimens) to the foaming solution led to a further increase in the foam adhesion to the struts due to the previously described binding effect of the polymer reports. Figure 8D(c,d) highlight a higher volume of material on the struts, well-interconnected with the surface of the strut and unmelted particles. The failure occurred at the metal–polymer interface, heterogeneously reporting adhesive and cohesive failure regions. Nonetheless, the foam appeared to be better organized when compared to the SF and SF_EW specimens. Once the electric field was applied (SFG_EW specimens), an additional increase in the apparent shear strength was registered. Reasonably, the electrically induced SF–G complexes were reconfigured in dipole formations with negative charges toward the anionic titanium surface, spatially organized along the electric field lines, as depicted in Figure 6B. The combined effect of SF nanofibril formation, the plate-like G-SF configuration, and the binding effect of G guaranteed an adhesive response more than twice higher compared to the SF or SF_EW specimens. Figure 8E(d) indeed highlights a more uniform and intimate connection of titanium particles and polymers with the conditions investigated previously.

Pull-out tests performed in wet conditions exhibited lower *τ_app_* values for all conditions compared to the dry state condition. Indeed, the *τ_app_* of SF, SF_EW, SFG, and SFG_EW is 23.3 kPa, 45.5 kPa, 22.8 kPa, and 78.0 kPa, respectively. As expected, this can be imputed to the plasticizing effect of water onto the SF or SFG structure. This behavior is also in agreement with the work of Maniglio et al. [51], where the authors showed a general lowering of all mechanical properties of the foams when they moved from a dry to a wet condition. It should be noted that the trend, depicted in Figure 8C, for the dry testing conditions, was no longer respected once the samples were soaked in water. In particular, the adhesion level of the SFG foams was comparable with the SF foams, thus suggesting that the binding role of gelatin, beneficial for a better structural organization of the foam, was lost, probably due to the swelling behavior and high water-uptake typical of gelatin [64]. Nevertheless, the improvement effect of electrowetting was still noticeable, either for the SF_EW or the SFG_EW conditions, as represented in Figure 8C. This behavior might still be related to the more organized and denser coating deposited at the metal–foam interface, where a spatial reorganization of protein chains can take place. Interestingly, in the hydrated state, the beneficial role of G in the blend was detectable only when the electric field was applied. The increasing adhesion, when compared to the SF_EW foams, might again be ascribed to the reorganization of the GSF complexes along with the deposition of SF-and its β-sheet reorganization onto the titanium surface.

It should also be noted that in both conditions, the reported interfacial shear strength was considerably lower than that of other works found in the literature, where SF hydrogels reported values in the MPa range [69,70,71,72]. This can be ascribed either to the high surface roughness of the struts to the structure of the polymeric scaffold, in a foam state. As investigated by Guo et al. [71], surface roughness strongly affected the degree of adhesion of a coating. The authors indeed reported that an optimum roughness *Ra* of 1 µm guaranteed the maximum adhesion strength of an SF coating on a Ti6Al4V substrate. This allowed for proper mechanical interlocking and low defect formation in the interfacial metal–polymer area. An increase in the surface roughness, typical of AM L-PBF Ti6Al4V lattices as in this case study [32], led to a consistent drop in the shear strength due to high defect formation at the interface. Additionally, highly porous systems such as foams not only exhibit lower mechanical properties but also cannot guarantee a conformal contact at the metal–polymer interface, as instead, a plasma-assisted or a pure EPD deposition can be generated [49,71].

### 3.5. Cytotoxicity Assessment

**Figure 9:**
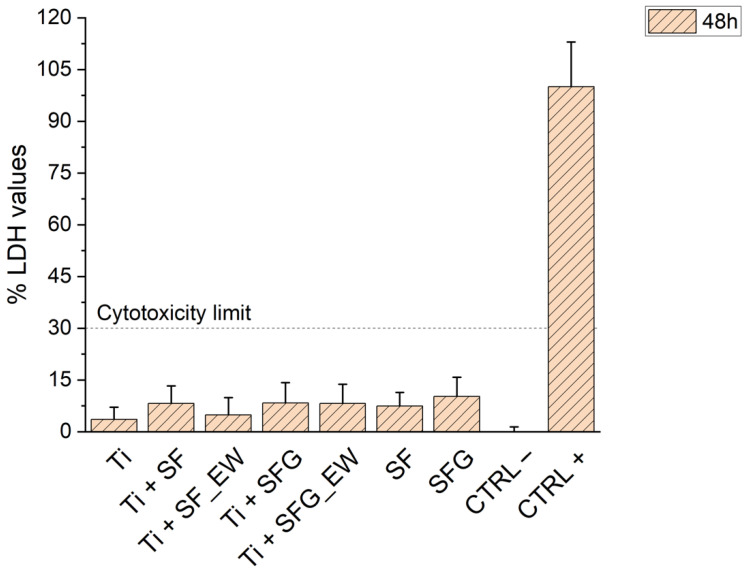
The bar plots reporting the %LDH values against the different conditions tested. Two controls, positive (CTRL+) and negative (CTRL−), were also reported. The evaluated conditions are the bare titanium cage (Ti), the four previously investigated composite conditions (herein classified as Ti + SF, Ti + SF_EW, Ti + SFG, and Ti + SFG_EW), and two stand-alone foams, namely one of pure silk fibroin (SF) and one of the blended compositions at a volume ratio of gelatin-silk fibroin 1:4 (SFG). The dashed line indicates the threshold limit below which the sample is not considered cytotoxic.

The LDH assay was carried out to investigate the potential cytotoxicity of the metal–polymer composite specimens as well as the SF and SFG foams. The histogram in Figure 9 reports the results of the assay after 48 h of incubation of the conditioned medium in contact with MRC5 cells. A sample was defined as cytotoxic, whereas the amount of LDH released into the medium was equal to or exceeded 30%. This threshold limit was set according to the EN ISO standards (European Standard EN ISO-10993-12:2004 and 10993-5:2009). As visible in Figure 9, all of the conditions tested were well below the 30% threshold limit, with average values generally below 10%. It is, therefore, possible to state that the manufacturing process (L-PBF combined with foaming or electrowetting techniques) as well as the involved materials (namely SF or G/SF blends) guarantees the production of noncytotoxic specimens.

## 4. Conclusions

We developed a novel multifunctional composite scaffold consisting of an LPBF Ti6Al4V lattice structure infilled with a silk fibroin or a silk fibroin/gelatin foam. To manufacture these hierarchical composite scaffolds, gas foaming of the protein solution onto the L-PBF lattice surface has been proposed. Additionally, to further assist the polymer adhesion on metal surfaces, the effect of an applied electric field during foaming (namely an electrowetting process) has been proposed.

For all four conditions, a porosity assessment was conducted, revealing comparable pore dimensions and morphologies. The main statistical differences were noted for the SFG and SFG_EW composites, which displayed a lower average pore size than the SF_EW specimens as well as a lower circularity. This might be ascribable to the binding role of gelatin and a spatial reorganization of silk fibroin–gelatin complexes under an electric field. The four composites exhibited different morphological features, imputed to the presence or absence of the electric field and the gelatin addition in the blend solution. The SF_EW composites showed nanofibril structures of silk fibroin, typical of the protein adhesion on metal surfaces. On the other hand, at the interface with the metal surfaces, SFG and SFG_EW specimens appeared to cover the outermost unmelted titanium particles more intimately. The pull-out tests confirmed the higher adhesion of SFG and SFG_EW composites in dry conditions. In the hydrated conditions, despite a general and expectable lowering of the apparent shear strength, the higher adhesive behavior of SFG_EW specimens was still confirmed. These preliminary results show the beneficial role of the application of an electric field during the gas foaming of the polymeric solution in promoting the favorable mechanisms of adhesion. The authors are aware that a deeper analysis of the driving mechanisms taking place at the metal–polymer interface should be carried out. Concerns might be related to the free cross-linked blend solution of gelatin and silk fibroin and its stability over time in a body fluid solution. Despite the cytotoxicity assessment revealing that all four conditions were well below the cytotoxicity limit, a more in-depth in-vitro characterization should be carried out. Specifically, the authors will leave for future works the evaluation of the bone marrow-derived human mesenchymal-stem cell differentiation toward an osteogenic phenotype. This will allow us to investigate how each condition can support cell spread and differentiation as well as long culture times. In parallel, degradation kinetics, in the presence and absence of enzymes, will also be evaluated. These results will be able to demonstrate the suitability of the proposed multifunctional composite hybrid system as a candidate for load-bearing bone implants with improved osteointegration.

## Figures and Tables

**Table 1:** The temperature of the sol–gel transition, °C, derived at the intersection of the moduli (G′ and G″) is reported for the pure gelatin solution (G) and the three-volume ratios investigated by G:SF, namely 1:2, 1:3, 1:4. The average values along with the standard deviation are reported. The ξ-potential, mV, of the diluted solutions of pure gelatin (G), the chosen mixture composition (G/SF 1:4), and pure silk fibroin (SF) are reported as the mean values along with the standard deviation, calculated in four replicas.

	G	G/SF 1:2	G/SF 1:3	G/SF 1:4	SF
Gelation Temperature [°C]	30.1 ± 1.0	21.5 ± 0.4	19.2 ± 0.1	19.1 ± 0.1	-
ξ-potential [mV]	+10.7 ± 0.7	-	-	+3.3 ± 0.1	−21.8 ± 1.4

**Table 2:** The main statistical parameters for the pore area, equivalent diameter, circularity, and aspect ratio. The reported parameters are the mean, standard deviation, median, and IQR value.

	Area [µm^2^]				Equivalent Diameter [µm]				Circularity				Aspect Ratio			
	*Mean*	*Std*	*Median*	*IQR*	*Mean*	*Std*	*Median*	*IQR*	*Mean*	*Std*	*Median*	*IQR*	*Mean*	*Std*	*Median*	*IQR*
**SF**	10,993	26,033	964	7664	77.7	89.4	35.0	79.0	0.56	0.23	0.57	0.36	2.03	1.17	1.61	1.06
**SF_EW**	17,607	41,434	1310	11,548	96.1	115.1	40.8	100.1	0.56	0.23	0.58	0.36	2.14	1.12	1.79	0.98
**SFG**	8852	16,601	2661	9059	81.1	68.6	58.2	82.5	0.59	0.18	0.62	0.31	1.91	0.99	1.59	0.88
**SFG_EW**	7245	12,656	957	8399	68.4	67.7	34.9	83.9	0.50	0.19	0.49	0.33	2.26	1.36	1.79	1.20

**Table 3:** Debonding force *F_deb_* [N] and apparent interfacial shear strength *τ_app_* [kPa] mean values along with their standard deviation for the four classes of specimens. Data were derived from the force–displacement pull-out curves for dry or wet conditions.

	Dry		Wet	
	*F_deb_* [N]	*τ_app_* [kPa]	*F_deb_* [N]	*τ_app_* [kPa]
**SF**	0.42 ± 0.14	76.7 ± 25.3	0.13 ± 0.04	23.3 ± 6.7
**SF_EW**	0.44 ± 0.06	79.8 ± 10.7	0.25 ± 0.03	45.5 ± 6.3
**SFG**	0.63 ± 0.02	115.1 ± 4.65	0.13 ± 0.03	22.8 ± 5.2
**SFG_EW**	0.89 ± 0.35	162.1 ± 63.5	0.43 ± 0.15	78.0 ± 28.2

## Data Availability

Not applicable.

## Supplementary Materials

The following supporting information can be downloaded at: https://www.mdpi.com/article/10.3390/ma15176156/s1, Table S1: Minimum, maximum values and, Skewness and Kurtosis values of the investigated porosity parameters (area A, equivalent diameter D_eq_, circularity C, and aspect ratio AR).
