# Supplementary material for: Hybrid Ti6Al4V/Silk Fibroin Composite for Load-Bearing Implants: A Hierarchical Multifunctional Cellular Scaffold
Source: Materials (Basel). 2022 Sep 5;15(17):6156. doi: 10.3390/ma15176156 (PMC9458142; doi:10.3390/ma15176156)
Supplement: Supplementary file 1 [file materials-15-06156-s001.zip › materials-1848862-supplementary.pdf]

Table S1. Minimum, maximum values and, Skewness and Kurtosis values of the investigated porosity parameters (area A, equivalent diameter D_{eq}, circularity C, and aspect ratio AR).

	**A [μm²]**				**D_{eq} [μm]**				**C**				**AR**			
	Min	*Max*	*Skewness*	*Kurtosis*	*Min*	*Max*	*Skewness*	*Kurtosis*	*Min*	*Max*	*Skewness*	*Kurtosis*	*Min*	*Max*	*Skewness*	*Kurtosis*
**SF**	201	231318	4.68	29.76	16	543	2.12	5.03	0.11	0.94	-0.17	-1.05	1.00	7.73	2.32	6.55
**SF_EW**	201	302065	4.21	21.86	16	620	2.05	4.47	0.08	1.00	-0.01	-0,90	1.06	6.92	2.07	4.63
**SFG**	201	139619	4.26	23.34	16	422	1.80	4.24	0.10	0.92	-0.34	-0.79	1.04	9.71	3.34	18.43
**SF SFG _EW**	200	57646	2.18	4.43	16	271	1.41	0.79	0.18	0.90	0.18	-1.12	1.06	8.75	2.30	6.36
